# Continuous Kidney Replacement Therapy With Adjusted Dialysate Sodium Concentration for Severe Hyponatremia in Beer Potomania: A Case Report

**DOI:** 10.7759/cureus.66834

**Published:** 2024-08-14

**Authors:** Masayuki Akatsuka, Hiroomi Tatsumi, Arata Osanami, Yuki Nakamura

**Affiliations:** 1 Department of Intensive Care Medicine, Sapporo Medical University School of Medicine, Sapporo, JPN; 2 Department of Cardiovascular, Renal and Metabolic Medicine, Sapporo Medical University School of Medicine, Sapporo, JPN; 3 Division of Clinical Engineering, Sapporo Medical University Hospital, Sapporo, JPN

**Keywords:** adjusted dialysate sodium concentration, beer potomania, continuous kidney replacement therapy, acute kidney injury, hyponatremia

## Abstract

Beer potomania is a condition characterized by severe hyponatremia in chronic alcoholics with poor nutritional intake. When complicated by acute kidney injury (AKI), it presents a significant management challenge. We report a case of a 32-year-old male with a history of alcoholism who presented with malaise, nausea, and vomiting. Laboratory tests revealed severe hyponatremia (serum sodium 104 mEq/L) and AKI. Conventional treatment approaches posed risks of overcorrection and osmotic demyelination syndrome (ODS). We implemented continuous kidney replacement therapy (CKRT) with meticulously adjusted dialysate sodium concentrations. This approach enabled gradual, controlled correction of serum sodium without precipitating ODS. The patient was successfully liberated from hemodialysis on the twelfth day of illness. Our findings highlight the potential of CKRT as an effective treatment modality for severe hyponatremia in beer potomania with AKI, offering a means of gradual sodium correction while addressing renal dysfunction. This case underscores the importance of tailored management strategies in complex clinical scenarios involving electrolyte imbalances and kidney injury.

## Introduction

Severe hyponatremia, characterized by serum sodium levels below 120 mEq/L, is an electrolyte imbalance that can lead to neurological complications and even fatalities if left untreated [1]. Beer potomania, a syndrome associated with excessive beer consumption and inadequate solute intake, is a known precipitant of profound hyponatremia [2-4]. Hyponatremia due to beer potomania can be caused by poor solute intake, dilution effect by the large volume of water from beer dilutes, and suppression of antidiuretic hormone [5]. Previous reports have highlighted the challenges in managing beer potomania-induced hyponatremia, particularly in cases complicated by acute kidney injury (AKI) [6-8]. Conventional treatment approaches, such as fluid restriction and hypertonic saline administration, might not be feasible or effective in such cases.

While intermittent kidney replacement therapy (IKRT) has been employed in some cases, it carries the risk of overcorrecting serum sodium levels, potentially leading to the development of osmotic demyelination syndrome (ODS) [9,10]. ODS can present with various neurological symptoms such as encephalopathy, seizures, Parkinsonian-like movement disorders, and locked-in syndrome [11-13]. The optimal management strategy for severe hyponatremia in beer potomania with concomitant AKI remains an area of uncertainty, with limited guidance on the appropriate rate of sodium correction and the potential role of continuous kidney replacement therapy (CKRT) in these complex cases.

Here, we report a case of severe hyponatremia in beer potomania complicated by AKI managed by CKRT with adjusted dialysate sodium concentrations, illustrating a novel approach to address this challenging clinical scenario.

## Case presentation

We present a case of a 32-year-old male who initially presented with the chief complaint of profound malaise accompanied by episodes of nausea and relentless vomiting. The patient’s medical history was punctuated by an extended battle with alcoholism, notably marked by recurrent cycles of alcohol dependence interspersed with sporadic hospitalizations for addiction treatment. The current episode started when the patient resumed excessive alcohol consumption, comprising 600 mL/day of whiskey and 240 g/day of ethanol equivalent.

While the patient had not previously demonstrated renal dysfunction or electrolyte abnormalities, a blood test conducted 15 days post-discharge from a previous hospitalization revealed severe renal dysfunction, coupled with hyponatremia (serum creatinine 17.1 mg/dL, serum blood urea nitrogen 129 mg/dL, and serum sodium 104 mEq/L).

A physical examination detected edema in both lower extremities and elevated blood pressure (178/110 mmHg), raising further concerns. Subsequent imaging studies, including chest radiography and plain computed tomography, revealed bilateral pleural effusion and renal enlargement, further heightening the clinical enigma.

Diagnosis eventually crystallized into a complex nexus encompassing hyponatremia, AKI, pleural effusion, rhabdomyolysis, liver dysfunction, and the inexorable specter of alcoholism. Notably, the steep drop in serum sodium concentration to 106 mEq/L upon admission cast a pall of urgency, necessitating renal replacement therapy and sodium correction.

**Figure 1:**
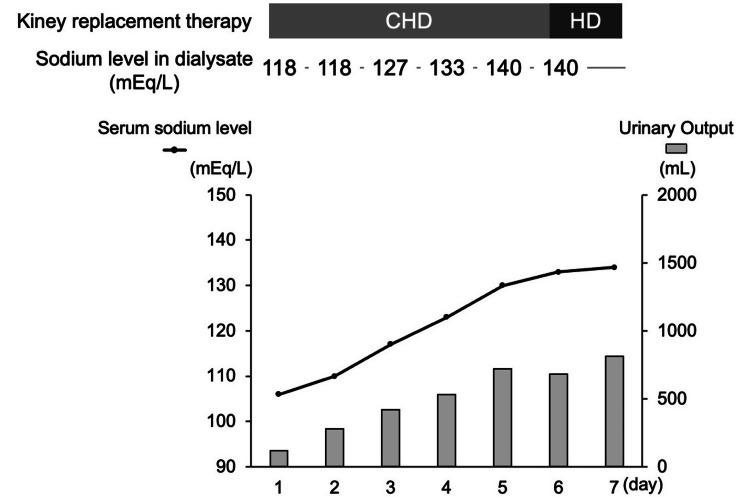
Post-hospitalization progress The daily sodium change was less than 8 mEq/L. The kidney replacement therapy was changed from CHD to HD on the 6th day. CHD, continuous hemodialysis; HD, hemodialysis

However, the conventional IKRT approach posed a threat of overcorrection, with potential consequences of ODS. To overcome this potential risk, we opted for CKRT with dialysate sodium concentrations meticulously adjusted using a 5% glucose solution. We have a specific method of adjusting the sodium concentration in the dialysate solution. When the 100 mL of 5% glucose solution was added to 2020 mL of dialysate, the sodium concentration was adjusted to 133 mEq/L; when the 200 mL of 5% glucose solution was added to 2020 mL of dialysate, the sodium concentration was adjusted to 127 mEq/L; and when the 300 mL of 5% glucose solution was added to 2020 mL of dialysate, the sodium concentration was adjusted to 118 mEq/L. We gradually changed the sodium concentration of the dialysate while checking the serum sodium concentration (Figure 1). This therapy led to successful liberation from hemodialysis on the twelfth day of illness. The patient transitioned to addiction-focused care on the nineteenth day of hospitalization.

## Discussion

In the intricate tapestry of medical complexities, our case illuminates two salient findings that underscore the nuanced interplay between therapeutic intervention and clinical outcomes. First, our management approach entailed the implementation of CKRT utilizing a dialysate solution with meticulously adjusted sodium concentrations, a pivotal intervention directed at addressing severe hyponatremia and AKI. Second, the meticulous sodium correction altered during the CKRT was marked by a gradual, controlled trajectory. This measured correction succeeded in raising the serum sodium level, thereby rectifying the primary electrolyte imbalance, and, critically, was executed without the onset of complications associated with ODS.

Our first notable finding pertains to using CKRT with a dialysate solution featuring meticulously adjusted sodium concentrations. This approach represents a departure from conventional management strategies and underscores the therapeutic rationale underpinning our intervention. Previous literature has documented the challenges associated with treating severe hyponatremia in beer intoxication [5,14-15]. Traditional methods, such as fluid restriction and hypertonic saline administration, should be carefully managed in such cases [16]. While IRRT has been employed, the inherent risk of rapid sodium correction poses a significant threat of precipitating ODS [17]. Our judicious implementation of CKRT with adjusted dialysate sodium concentrations aimed to circumvent these limitations, thereby highlighting a novel therapeutic venue tailored to the intricacies of our case.

The second important finding of this study is that the gradual and controlled trajectory of sodium correction achieved through our CKRT approach warrants further examination. The measured elevation of serum sodium levels, accomplished without precipitating complications associated with ODS, stands as a testament to the efficacy of our strategy. Existing literature underscores the perils of rapid sodium correction, with well-documented cases of ODS arising from injudicious management [18]. Our findings, however, challenge the prevailing notion that severe hyponatremia necessitates an expeditious correction, particularly in the context of beer potomania complicated with AKI.

The interpretation and clinical implications of this care report are multifaceted. First, our findings underscore the necessity for a nuanced, case-specific approach to the management of severe hyponatremia in the context of beer potomania and AKI. The complexity of this clinical scenario demands a departure from conventional treatment modalities, as evidenced by the limitations of fluid restriction, hypertonic saline, and IKRT. Second, our study highlights the potential utility of CKRT as a viable therapeutic modality in such cases, offering a means of gradual sodium correction while addressing renal dysfunction. This approach mitigates the risk of ODS and facilitates a comprehensive management strategy tailored to the patient's underlying pathophysiology.

## Conclusions

Our case report illuminates a novel therapeutic approach to manage severe hyponatremia in beer potomania complicated by AKI. The judicious implementation of CKRT with adjusted dialysate sodium concentrations enabled a gradual, controlled correction of serum sodium levels, rectifying the primary electrolyte imbalance while circumventing the perils of rapid overcorrection and ODS. These findings underscore the importance of tailored, case-specific management strategies in complex clinical scenarios and highlight the potential utility of CKRT as a valuable addition to the therapeutic armamentarium. As we continue to navigate the intricacies of rare clinical entities, such as beer potomania, the insights gleaned from this case will undoubtedly inform future management approaches, ultimately improving patient outcomes and advancing the frontiers of medical knowledge.
